# High-Performance Electrochromic Devices Based on Size-Controlled 2D WO_3_ Nanosheets Prepared Using the Intercalation Method

**DOI:** 10.3390/ma17010041

**Published:** 2023-12-21

**Authors:** Cheng-Ai Li, Boemjin Ko, Kwang-Hyun Park, Jae-Gyu Ahn, Taeyoung Park, Dong-Ju Lee, Sung-Ho Song

**Affiliations:** 1 Division of Advanced Materials Engineering, Center for Advanced Powder Materials and Parts, Kongju National University, Cheonan 32588, Republic of Korea; lca12@kongju.ac.kr (C.-A.L.); qjawls5004@smail.kongju.ac.kr (B.K.); recite14@kongju.ac.kr (K.-H.P.); a20220102@smail.kongju.ac.kr (J.-G.A.); a20230134@smail.kongju.ac.kr (T.P.); 2 Department of Advanced Materials Engineering, Chungbuk National University, Chungdae-ro 1, Seowon-gu, Cheongju 28644, Republic of Korea

**Keywords:** tungsten trioxide, nanosheet, intercalation, electrochromic device

## Abstract

It is difficult to obtain ultrathin two-dimensional (2D) tungsten trioxide (WO_3_) nanosheets through direct exfoliation from bulk WO_3_ in solution due to the strong bonding between interlayers. Herein, WO_3_ nanosheets with controllable sizes were synthesized via K^+^ intercalation and the exfoliation of WO_3_ powder using sonication and temperature. Because of the intercalation and expansion in the interlayer distance, the intercalated WO_3_ could be successfully exfoliated to produce a large quantity of individual 2D WO_3_ nanosheets in *N*-methyl-2-pyrrolidone under sonication. The exfoliated ultrathin WO_3_ nanosheets exhibited better electrochromic performance in an electrochromic device than WO_3_ powder and exfoliated WO_3_ without intercalation. In particular, the prepared small WO_3_ nanosheets exhibited excellent electrochromic properties with a large optical modulation of 41.78% at 700 nm and fast switching behavior times of 9.2 s for bleaching and 10.5 s for coloring. Furthermore, after 1000 cycles, the small WO_3_ nanosheets still maintained 86% of their initial performance.

## 1. Introduction

Electrochromic (EC) materials have attracted considerable interest in various applications, including transparent displays, smart windows, and automotive mirrors, owing to their ability to efficiently adjust optical properties, such as transmittance, reflectance, and absorbance with low power consumption [1,2,3,4]. The optical properties of EC materials are reversibly altered through the injection and extraction of ions and electrons into the EC layer under a low applied voltage between the anode and the cathode. Among a number of EC materials, tungsten trioxide (WO_3_) has been widely studied due to its good EC performance and electrochemical stability [5,6]. To further enhance the EC performance of WO_3_, substantial efforts have been committed to producing nanostructured WO_3_ [7,8,9]. In particular, in contrast to bulk WO_3_, two-dimensional (2D) WO_3_ nanosheets have been found to exhibit enhanced optical contrast and durability due to the expanded active surface area and facilitated ion penetration within EC materials [10,11]. Various techniques are used to produce 2D WO_3_ materials, achievable through two synthetic strategies: the top-down and the bottom-up approach [10,12,13,14,15,16,17]. The bottom-up methods aim to produce 2D WO_3_ from smaller precursor molecules, whereas the top-down approach involves the direct exfoliation of bulk precursors. Exfoliation emerges as a highly efficient approach for producing a multitude of ultrathin 2D nanosheets from the bulk source. However, because of the strong covalent interaction between the layers of monoclinic WO_3_, the synthesis of ultrathin 2D nanosheets from bulk WO_3_ using a typical liquid-phase exfoliation method is still remarkably challenge. Only a few studies on the production of ultrathin 2D WO_3_ nanosheets with liquid-phase exfoliation have been reported to date. This can be achieved through methods such as the direct exfoliation of bulk WO_3_, exfoliation and oxidation from tungsten disulfide (WS_2_), and the exfoliation of hydrated WO_3_ [11,15,16,18,19]. Guan et al. achieved the direct exfoliation of WO_3_ powder into thin nanosheets by coating the surface of WO_3_ with bovine serum albumin (BSA) [18]. In an acidic environment, the -NH_2_ functional groups of BSA establish strong bonds with WO_3_ via electrostatic interaction, thereby considerably improving the exfoliation process during sonication. However, due to strong binding in WO_3_, prolonged processing times (sonication for 48 h) are required, and the residual presence of BSA may restrict their subsequent applications. In addition, Azam et al. reported a solution-phase method for synthesizing 2D WO_3_ nanosheets, involving the oxidation of 2D WS_2_ nanosheets obtained through the exfoliation of bulk WS_2_ powder [11]. Typically, obtaining fully oxidized WO_3_ nanosheeets requires distinct exfoliation and oxidation steps, making these processes more time-consuming compared to single-step methods. Subsequent investigations aimed to generate WO_3_ intercalation compounds with the goal of producing WO_3_ nanosheets without the need for additional oxidation processes. The layered WO_3_ hydrates can serve as effective precursors for the preparation of ultrathin WO_3_ nanosheets [15,16,20]. Hydrated WO_3_ is typically manufactured on a nanometer scale, resulting in exfoliated nanosheets with small lateral sizes (less than 500 nm). To our knowledge, no method has been reported for directly producing large 2D WO_3_ nanosheets from bulk WO_3_ without the hydration step. In order to prepare 2D nanosheets by exfoliating bulk WO_3_, an intercalation process was utilized to weaken the interlayer binding forces in the materials.

Intercalation occurs when molecules or ions are incorporated into the layers of bulk source materials, the interlayer distance is expanded, and the binding interactions between the adjacent layers are weakened. Depending on the bulk source materials, the intercalating species, and its concentration, a variety of different intercalation compounds with intriguing properties can be used for synthesis [21,22]. The exfoliation method following intercalation isolates the atomic layers directly from the bulk materials. Importantly, an appropriate solvent plays a crucial role in the intercalation-based exfoliation and subsequent processing of layered materials [21,23,24]. The solvent properties, such as polarity and surface energy (surface tension), significantly impact the quality of the final products. The solvent polarity influences intercalation efficiency by determining the dissolution or solvation of the intercalation compounds. Additionally, the surface energy (or surface tension) of the solvent is essential for effective exfoliation.

Here, we report a facile method to synthesize 2D WO_3_ nanosheets with controllable sizes through the intercalation of K^+^ into bulk WO_3_ powder and exfoliation using *N*-methyl-2-pyrrolidone (NMP) with similar surface tension. In this method, lamellar intercalated WO_3_ is initially prepared by intercalating K^+^ into bulk WO_3_. Subsequently, WO_3_ nanosheets with various sizes and thicknesses below 10 nm are exfoliated by carefully controlling the exfoliation conditions. The synthesized WO_3_ nanosheets are then incorporated into an electrochromic device, and the impact of the WO_3_ nanosheet size on electrochromic performance is examined. The electrochromic device using small nanosheets demonstrates a broad optical contrast, a rapid coloration/bleaching response, and robust cycling stability.

## 2. Materials and Methods

### 2.1. Intercalation and Exfoliation of WO_3_ Nanosheets

**Figure 1:**
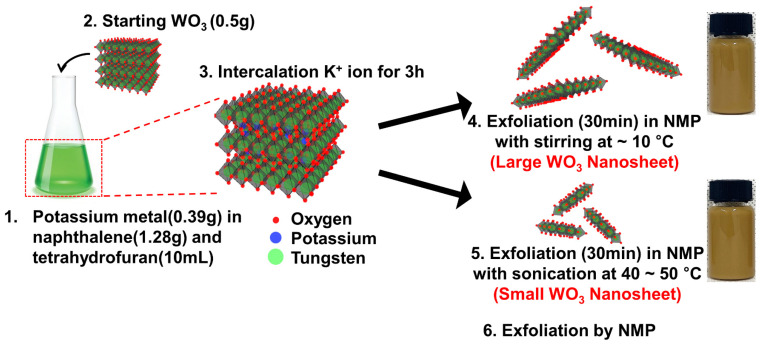
Schematic illustration of the intercalation and exfoliation process used for the synthesis of 2D WO_3_ nanosheets from bulk powder.

A homogenously ground mixture of WO_3_ powder (0.5 g; Sigma-Aldrich, Milwaukee, WI, USA), potassium metal (0.39 g; Sigma-Aldrich, Milwaukee, WI, USA), naphthalene (1.28 g; Sigma-Aldrich, Milwaukee, WI, USA), and tetrahydrofuran (10 mL; Sigma-Aldrich, Milwaukee, WI, USA) was reacted for 3 h. During the reaction, K^+^ intercalated into layers of bulk WO_3_ and expanded the bulk of WO_3_. The intercalated compounds of WO_3_ were stirred and sonicated in NMP (30 mL; Sigma-Aldrich, Milwaukee, WI, USA) for 30 min at 10 °C and 40~50 °C for the exfoliation of large and small WO_3_ nanosheets, respectively. In theory, increased energy has the potential to enhance the fragmentation of atomic layers, leading to a significant reduction in the lateral size of the nanosheets [25]. Exfoliation achieved through gentle stirring at a low temperature can prevent in-plane damage, resulting in the formation of larger nanosheets in the solution [26]. Following centrifugation at 1500 rpm for 30 min, a uniformly dispersed yellowish supernatant containing exfoliated WO_3_ nanosheets was collected by discarding the precipitate of unexfoliated WO_3_ nanosheets. For comparison, the exfoliated WO_3_ powder in NMP without intercalation was also prepared. The corresponding preparation process is illustrated in Figure 1.

### 2.2. Fabrication of Electrochromic Devices (ECDs)

**Figure 2:**
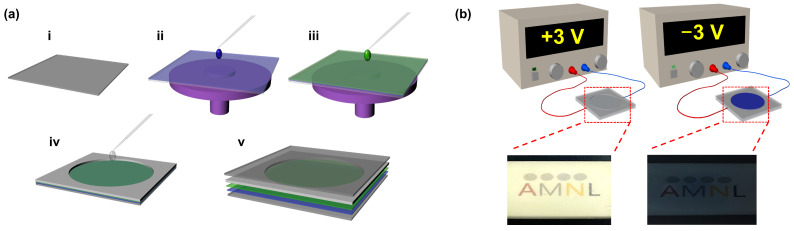
Schematic of (**a**) the fabrication of ECD and (**b**) the ECD performance test.

Bare indium tin oxide (ITO)-coated glass, used as an electrode, was cleaned via sonication in ethanol, acetone, and isopropanol for 15 min, respectively. Thin films composed of WO_3_ ultrathin nanosheets were then spin-coated on the cleaned ITO/glass at 4000 rpm for 60 s and dried at 120 °C for 10 min. Subsequently, 1 M lithium perchlorate (LiClO_4_) in propylene carbonate (PC), used as the electrolyte, was injected into an adhesive spacer between two ITO electrodes. Finally, another ITO electrode was placed on top of the spacer, and then the device was sealed (Figure 2a). The prepared ECDs with the structure of ITO/WO_3_/LiClO_4_ + PC/ITO were used to evaluate the performance of the ultrathin WO_3_ nanosheets.

### 2.3. Characterization of WO_3_ Nanosheets

The morphologies and structures were examined via scanning electron microscopy (SEM, MiRA3-LMH, Tescan, Brno, Czech Republic), transmission electron microscopy (TEM, JEM-F200, JEOL, Tokyo, Japan), and atomic force microscopy (AFM) in tapping mode under ambient conditions using the X2-70 system from Park Systems Corp, Suwon, Republic of Korea. The crystal structure of the prepared WO_3_ films was investigated by utilizing X-ray diffraction (XRD) with the MiniFlex 600 instrument from Rigaku, Tokyo, Japan and employing a Cu radiation source at a scan rate of 5° min^−1^. Raman spectra were measured using a Raman spectrometer (LabRAM HR, HORIBA, Longjumeau, France) from 200 to 900 cm^−1^ with 325 nm laser excitation. The samples’ chemical compositions were examined using X-ray photoelectron spectroscopy (XPS) with a K-Alpha X-ray photoelectron spectrometer from Thermo Scientific, Loughborough, UK.

### 2.4. ECD Performance Test

Electrochemical measurements for the EC device were conducted using a BioLogic SP-150 Potentiostat (BioLogic Science Instruments, Seyssinet-Pariset, France) within a three-electrode system, where the working electrode was WO_3_ films on the ITO, the reference electrode was Ag/AgCl (1 M KCl), and the counter electrode was graphite. The cyclic voltammetry (CV) curves were obtained by applying a potential range from +1.0 V to −1.0 V at a scan rate of 20 mV s^−1^. Additionally, a chronoamperometry test was performed, as shown in Figure 2b, using an operating voltage window between +3.0 V and −3.0 V with a 30 s interval. Furthermore, UV-Vis spectroscopy (UV-3600, Shimadzu, Kyoto, Japan) was used for measuring the optical color contrast and switching response time of the ECD, with an operating voltage ranging from +3.0 V to −3.0 V.

## 3. Results and Discussion

**Figure 3:**
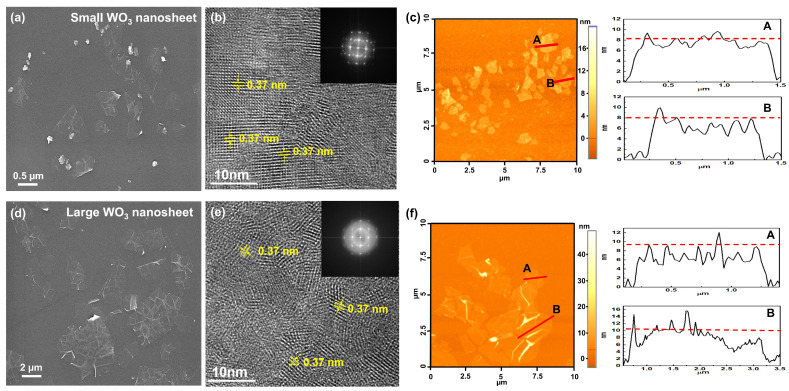
(**a**) SEM image, (**b**) HRTEM image, and (**c**) AFM image of small WO_3_ nanosheets. (**d**) SEM image, (**e**) HRTEM image, and (**f**) AFM image of large WO_3_ nanosheets.

In the experimental process, WO_3_ nanosheets were synthesized from bulk WO_3_ using a two-step procedure. This involved initially intercalating WO_3_ with K^+^ ions, followed by the subsequent exfoliation of layered intercalated compounds into nanosheets, as illustrated in Figure 1. The morphology of exfoliated WO_3_ nanosheets with controllable sizes was investigated via SEM, TEM, and AFM. SEM images showed the successful production of WO_3_ nanosheets with a lamellar structure (Figure 3a,d). However, there were more wrinkles between the structures in the large WO_3_ nanosheets compared with the small nanosheets. The size of the small WO_3_ nanosheets was sub-1 μm, whereas the size of the large WO_3_ nanosheets was greater than 1 μm (Figure S1). During the sonication process, higher temperatures in the sonication bath promote the exfoliation and reduce the average size of the nanosheets, due to the increased sonic pressure at elevated temperatures [27,28]. Figure 3b,e show high-resolution transmission electron microscopy (HRTEM) images of individual small and large WO_3_ nanosheets, respectively. HRTEM images of the nanosheets display distinct lattice fringes, indicating their remarkable crystal structure. All the clear lattice spacings were 0.37 nm, corresponding to the (002) plane of monoclinic WO_3_ [29,30]. In addition, the selected area electron diffraction patterns of the small and large nanosheets illustrate their single crystal nature, respectively (inset of Figure 3b,e). AFM was used to further analyze the thickness of the WO_3_ nanosheets. As can be seen from Figure 3c and Figure S2, the thickness of the small WO_3_ nanosheets was about 8 nm, corresponding to the sub-layer structure of the WO_3_. The thickness of the large WO_3_ nanosheets was about 10 nm, and there were more wrinkles between the structures (Figure 3f and Figure S3), which was consistent with the SEM analysis. Using the intercalation and exfoliation of bulk WO_3_, the majority of nanosheets were found to exhibit a fundamental thickness below 10 nm, approximately 10 layers of monoclinic WO_3_ unit cells [16].

**Figure 4:**
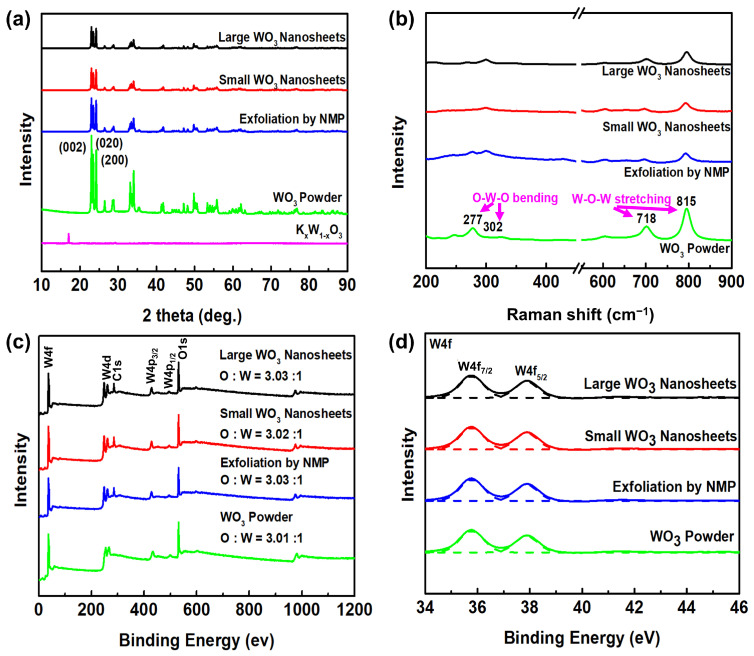
(**a**) XRD patterns, (**b**) Raman spectra, (**c**) wide-scan XPS spectra, and (**d**) high-resolution XPS spectra of W 4f of the exfoliated WO_3_ and bulk WO_3_ powder.

The XRD pattern analysis of nanosheets also confirmed the crystalline structure. As presented in Figure 4a, the crystalline structures of the prepared large WO_3_ nanosheets, small WO_3_ nanosheets, NMP exfoliation, and WO_3_ powder were determined through XRD analysis. For comparison, the XRD pattern of intercalated compound of WO_3_ (K_x_W_1-x_O_3_) used for exfoliation was also studied. In the XRD pattern of WO_3_ powder, there were well-defined peaks located at 23.1°, 23.7°, and 24.2°, corresponding to the (002), (020), and (200) planes of the WO_3_ monoclinic structure (JCPDS 43-1035), respectively [11,31]. All the peaks of the exfoliated WO_3_ nanosheets were quite similar to the monoclinic structure of WO_3_ powder, indicating that the exfoliation had no effect on the crystalline structure of WO_3_ in the nanosheets. In contrast, the peak positions of intercalated WO_3_ shifted toward smaller angle values compared to the WO_3_ powder due to the intercalation and expansion of the interlayer distance. Therefore, intercalated WO_3_ can be successfully exfoliated to produce a large quantity of individual 2D WO_3_ nanosheets via sonication in NMP.

Raman spectroscopy is additionally utilized to examine the crystalline structure of the exfoliated products. The Raman spectra of (a) large WO_3_ nanosheets, (b) small WO_3_ nanosheets, (c) NMP exfoliation, and (d) WO_3_ powder are shown in Figure 4b and Figure S4. In the Raman spectrum of WO_3_ (curve d), the peaks at 277 and 302 cm^−1^ were assigned to the bending modes of O-W-O, while the peaks at 718 and 815 cm^−1^ were identified as the stretching modes of W-O-W of monoclinic phase [11,31,32]. The Raman spectra of the nanosheets showed all the characteristic peaks of WO_3_ in curves a and b, demonstrating that the nanosheets were successfully exfoliated from the WO_3_ powder. It should be noted that the band intensity of nanosheets at 277 cm^−1^ decreased compared with the WO_3_ powder. This indicates a substantial reduction in the layer number of exfoliated nanosheets, which was consistent with the remarkable decrease in the XRD intensity.

In order to further study the components of the nanosheets, XPS characterizations were carried out. Figure 4c,d shows the XPS survey and W 4f of the prepared samples. In the survey scan spectra of the prepared WO_3_ samples, the presence of W and O was evident. The peaks and the atomic ratios of O to W in the exfoliated WO_3_ nanosheets closely resemble those of the WO_3_ powder, suggesting that the exfoliation process had no impact on the WO_3_ components in the nanosheets. The W 4f core level spin split into two energy states, namely W 4f_7/2_ and W 4f_5/2_, and the peak positions of these two energy levels thus were located at 35.2 and 37.3 eV, respectively. These peak positions were aligned with those reported in the literature, confirming that the nanosheets of WO_3_ existed in the highest oxidation state (W^6+^) [11,31,33]. XPS analysis, combined with XRD and Raman analyses, verified that the nanosheets were successfully exfoliated with controllable sizes. Hence, it was anticipated that the electrochromic performance of the exfoliated WO_3_ nanosheets would surpass that of bulk WO_3_.

The electrochemical properties of the WO_3_ nanosheets were analyzed using the CV method. Figure S5 presents the CV curves of these deposited films carried out in a potential range of −1.0 to 1.0 V at a scan rate of 20 mV s^−1^. The CV curves show cathodic and anodic currents due to the intercalation and extraction processes as follows [5,6,7,11,32,34]:

(1) $${\mathrm{WO}}_{3}\;(\mathrm{bleached}\;\mathrm{state})\;+\;{\mathrm{xLi}}^{+}\;+\;{\mathrm{xe}}^{-}\;↔\;{\mathrm{Li}}_{x}{\mathrm{WO}}_{3}\;(\mathrm{colored}\;\mathrm{state})$$

where Li^+^, in this study, denotes the ions in the polymer lithium perchlorate electrolyte. By applying a negative potential, charges and Li ions were inserted into the WO_3_ films, which rapidly changed the color of the film to dark blue (colored state). In contrast, with the application of a positive potential, the charges and Li ions were extracted from the WO_3_ films and showed a milky white color (bleached state). The shapes of the CV curves of the prepared WO_3_ samples were very similar and the potentials of the redox peaks did not exhibit significant differences (Figure S5). Nevertheless, the anodic and cathodic peak currents of the WO_3_ nanosheets were much higher than those of the WO_3_ powder and exfoliation using NMP, confirming the higher electrochemical activity of Li^+^ in the nanosheets. The utilization of WO_3_ nanosheets in the devices enabled greater charge insertion in each coloring-bleaching cycle and was expected to necessitate shorter coloration times. The increase in the cathodic and anodic peak currents was an indication of an increasing amount of active mass deposited on the substrate, due to the larger surface area of the ultrathin nanosheets.

**Figure 5:**
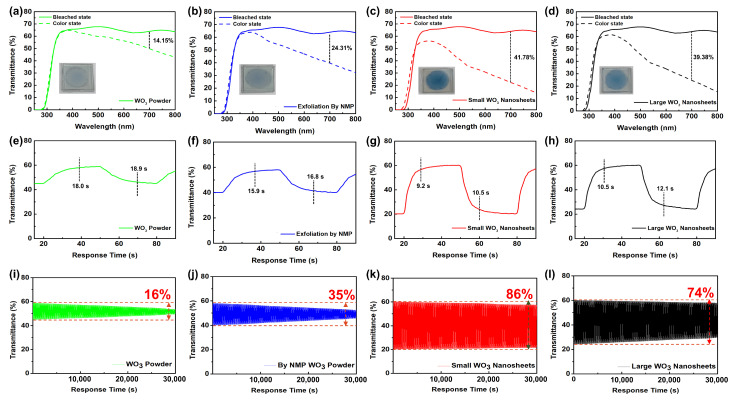
Transmittance spectra and digital images of ECDs using (**a**) WO_3_ powder, (**b**) NMP exfoliation, (**c**) small WO_3_ nanosheets, and (**d**) large WO_3_ nanosheets. Transmittance switching response times of (**e**) WO_3_ powder, (**f**) NMP exfoliation, (**g**) small WO_3_ nanosheets, and (**h**) large WO_3_ nanosheets. Stability test of ECDs using (**i**) WO_3_ powder, (**j**) NMP exfoliation, (**k**) small WO_3_ nanosheets, and (**l**) large WO_3_ nanosheets.

In addition to evaluating the electrochemical performance, the electrochromic properties of the WO_3_ nanosheets were further studied. The transmittance spectra of WO_3_ powder, NMP exfoliation, small WO_3_ nanosheets, and large WO_3_ nanosheets in the bleached and colored states are presented in Figure 5a–d following the application of ±3.0 V across the two ITO electrodes. The WO_3_-based ECD demonstrated reversible color changes across the entire surface, changing between the bleached state at 3.0 V and the colored state at −3.0 V (Figure 2b). The ECDs exhibited notable variations in transmittance within the visible light spectrum range of 350 to 800 nm. The maximum optical contrast of the small WO_3_ nanosheets was found to be 41.78% at 700 nm (Figure 5c), which was relatively high compared to the WO_3_ powder (14.15%), NMP exfoliation (24.31%), and large WO_3_ nanosheets (39.38%). Moreover, the distinct observation of the blue color was evident in the ECD by utilizing small WO_3_ nanosheets, as shown in the inset of Figure 5c. This suggests that the electrochromic properties of the small WO_3_ nanosheet are reasonably good within the visible light spectrum range. Ultimately, the use of smaller WO_3_ nanosheets can enhance the modulation between the bleached and colored state, where a large optical modulation is highly desired in ECDs. In addition, the transmittance at 700 nm under the alternating potential between +3.0 and −3.0 V for 30 s is displayed, and the corresponding times for bleaching and coloring, defined as the duration to achieve 90% of the final change in transmittance, are recorded in Figure 5e–h and Figure S6. Fast switching speeds of 9.2 s for bleaching and 10.5 s for coloring were obtained using small WO_3_ nanosheets; these times were faster compared to those of the WO_3_ powder (18.0 s for bleaching and 18.9 s for coloring), NMP exfoliation (15.9 s for bleaching and 16.8 s for coloring), and large WO_3_ nanosheets (10.5 s for bleaching and 12.1 s for coloring). The ECD using small WO_3_ nanosheets exhibited a significant optical contrast and reduced switching time, indicating the facile intercalation of Li^+^ ions into the smaller WO_3_ nanosheets (electrochromic layer) from the electrolyte. The coloration time of the small nanosheets was comparable to the times of the previously reported WO_3_ nanosheets [11,20,35,36] (Table S1). Unfortunately, the bleaching time of the small nanosheets was a little higher than the times of the previously reported WO_3_ nanosheets [11,20,35,36] (Table S1).

Another important aspect of electrochromic performance is reversibility. Coloring–bleaching cycling performance was also evaluated via transmittance at 700 nm under an alternating potential of ±3.0 V (Figure 5i–l). As shown in Figure 5k, there was no obvious degradation in the transmittance modulation during the cycling process of 5000 s and a reversibility rate of 86% was found for 30,000 s (1000 cycles), demonstrating the fine electrochromic stability of the small WO_3_ nanosheets. However, reversibility rates of 16%, 35%, and 74% were observed using WO_3_ powder, exfoliated WO_3_ without intercalation, and large WO_3_ nanosheets in ECDs, respectively. The excellent cycling durability of the small nanosheets is comparable to the times of the previously reported WO_3_ nanosheets [11,18,20,35] (Table S1).

## 4. Conclusions

Ultrathin WO_3_ nanosheets with varying sizes were synthesized using the intercalation method. The small WO_3_ nanosheets were smaller than 1 μm and the large WO_3_ ranged from 2 to 5 μm. The effects of nanosheet size on the EC properties of WO_3_ were elucidated. The obtained high optical contrast was attributed to the high optical modulation between the colored and bleached states of the small WO_3_ nanosheets. In addition, the smaller nanosheets showed a decrease in coloring and bleaching times compared with the WO_3_ powder, NMP exfoliation, and large WO_3_ nanosheets, respectively. Moreover, the small WO_3_ nanosheets exhibited reversibility of 86%.

## Data Availability

All the data are contained within the article and the Supplementary Materials.

## Supplementary Materials

The following supporting information can be downloaded at: https://www.mdpi.com/article/10.3390/ma17010041/s1. Figure S1: Size distribution of (a) small synthesized WO_3_ nanosheets and (b) large WO_3_ nanosheets; Figure S2: AFM image of small WO_3_ nanosheets; Figure S3: AFM image of large WO_3_ nanosheets; Figure S4: Magnified Raman spectra of (a) large WO_3_ nanosheets, (b) small WO_3_ nanosheets, (c) NMP exfoliation, and (d) WO_3_ powder; Figure S5: CV curves of the prepared WO_3_ samples at 20 mV s^−1^ in 1 M LiClO_4_ containing PC; Figure S6: Magnified transmittance modulation of (a) WO_3_ powder, (b) NMP exfoliation, (c) small WO_3_ nanosheets, and (d) large WO_3_ nanosheets during the cycling process between 14,000 and 15,000 s; Table S1: Performance comparison of EC devices made up of WO_3_ nanosheets.
