# Supplementary material for: High-Performance Electrochromic Devices Based on Size-Controlled 2D WO3 Nanosheets Prepared Using the Intercalation Method
Source: Materials (Basel). 2023 Dec 21;17(1):41. doi: 10.3390/ma17010041 (PMC10780075; doi:10.3390/ma17010041)
Supplement: Supplementary file 1 [file materials-17-00041-s001.zip › materials-2762298-supplementary.pdf]

Supplementary Materials

High-Performance Electrochromic Devices Based on Size-Controlled 2D WO_3 Nanosheets Prepared by Intercalation Method

Cheng Ai Li [1,†], Boemjin Ko [1,†], Kwang Hyun Park [1], Jae Gyu Ahn [1], Taeyoung Park [1], Dong Ju Lee [2,*] and Sung Ho Song [1,*]

[1] Division of Advanced Materials Engineering and Center for Advanced Powder Materials and Parts, Kongju National University, Cheonan, Chungnam 32588, Republic of Korea

[2] Department of Advanced Materials Engineering, Chungbuk National University, Chungdae-ro 1, Seowon-Gu, Cheongju, 28644, Republic of Koera

[†] These authors contributed equally to this work.

[*]Correspondence: shsong805@kongju.ac.kr (S. H. Song); dongjulee@chungbuk.ac.kr (D. J. Lee)

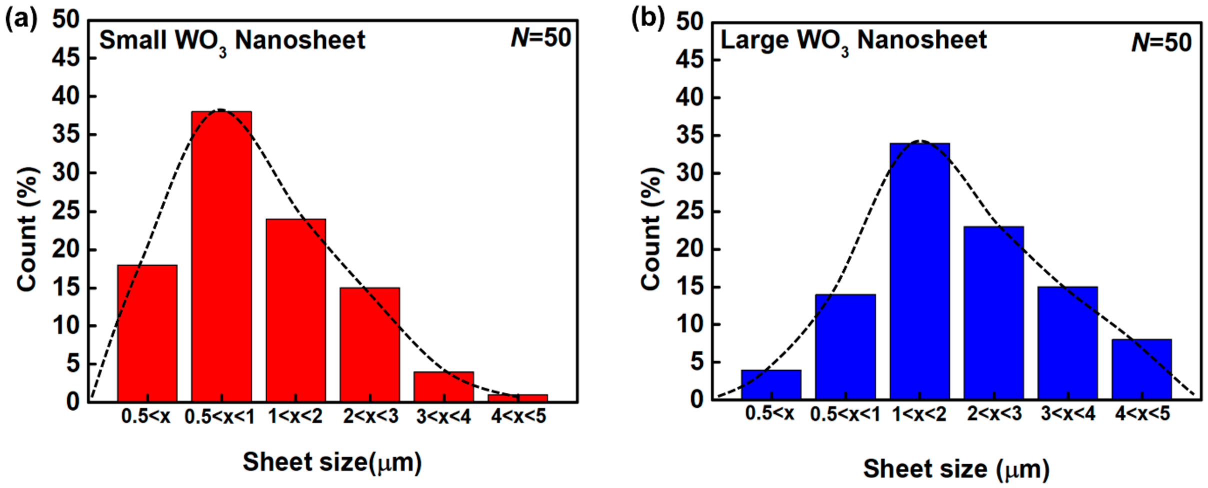

Figure S1. Size distribution of synthesized (a) Small WO_3 nanosheets and (b) Large WO_3 nanosheets.

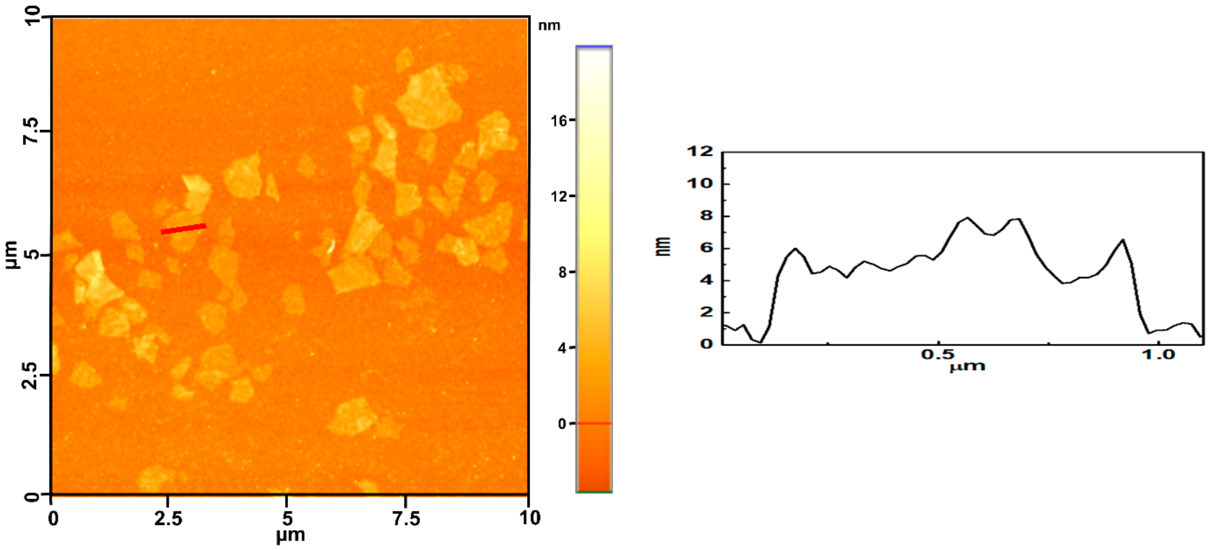

Figure S2. AFM image of small WO_3 nanosheets.

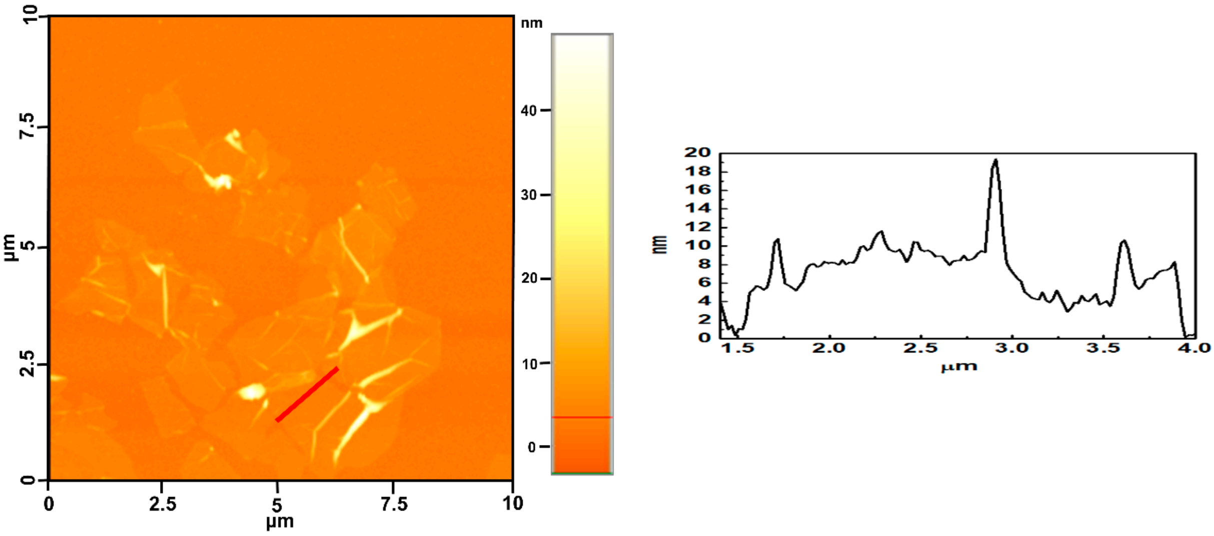

Figure S3. AFM image of large WO_3 nanosheets.

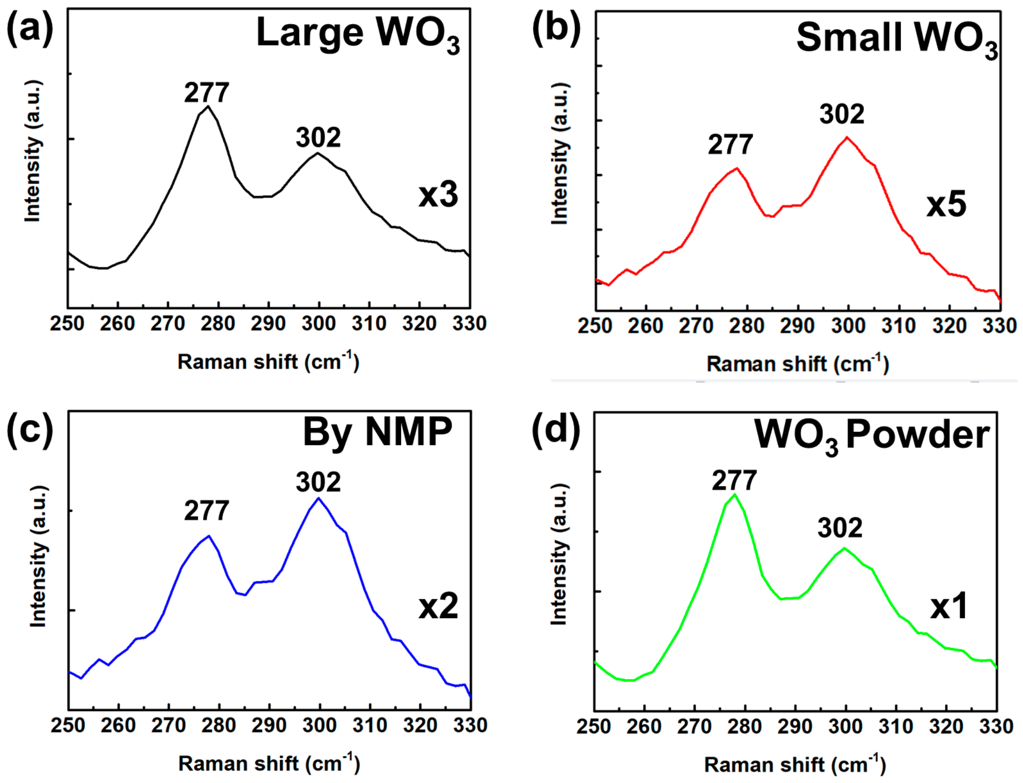

Figure S4. Magnified Raman spectra of (a) Large WO_3 nanosheet, (b) Small WO_3 nanosheets, (c) Exfoliated by NMP, and (d) WO_3 powder.

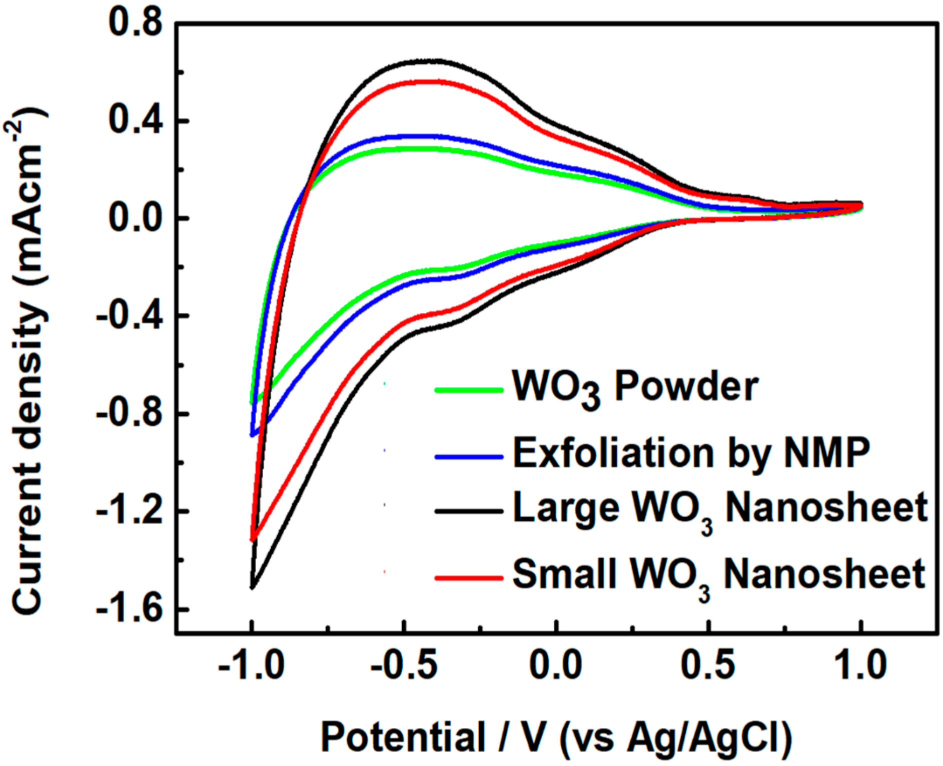

Figure S5. CV curves of the prepared WO_3 samples at 20 mV s^{-1} in 1 M $LiClO_4$ containing PC.

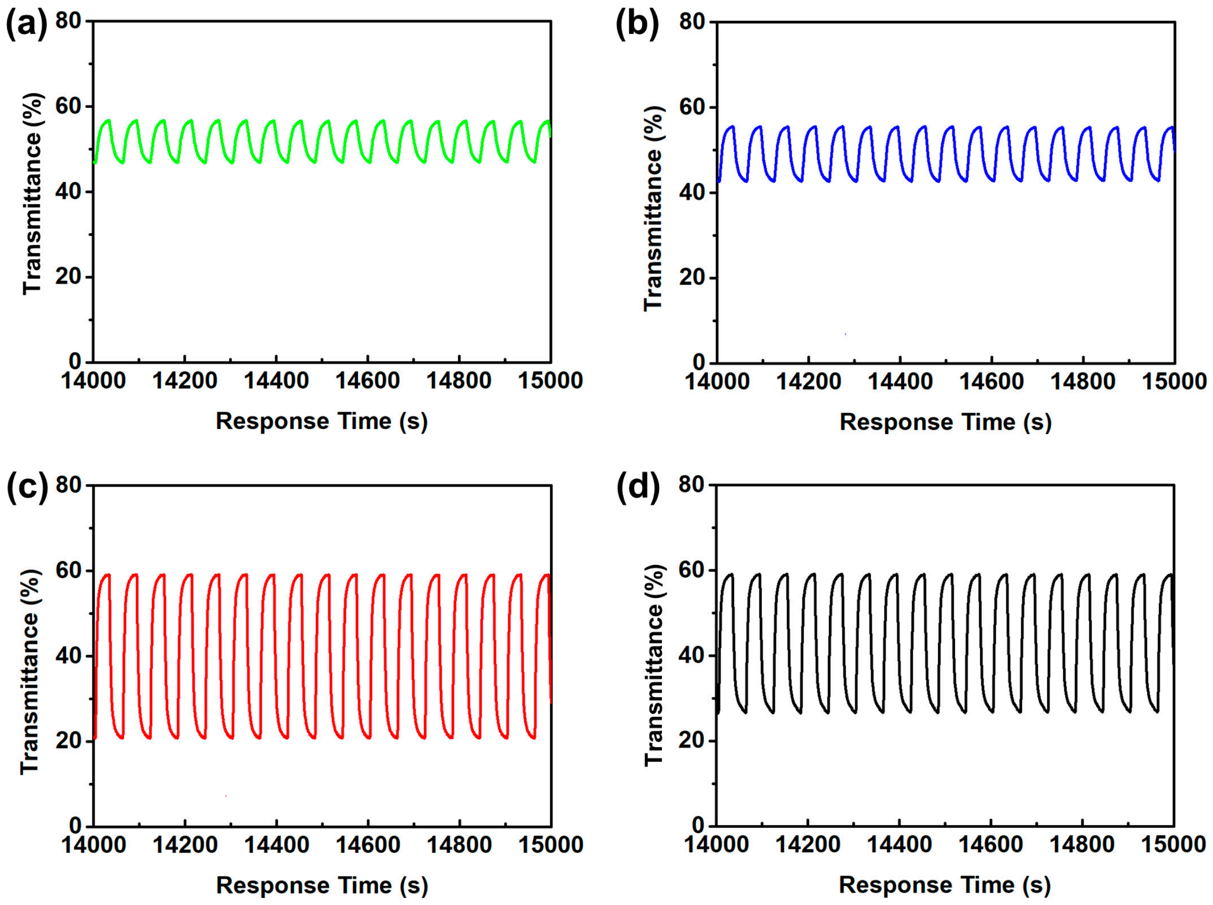

Figure S6. Magnified transmittance modulation of (a) WO_3 powder, (b) Exfoliated by NMP, (c) Small WO_3 nanosheets, and (d) Large WO_3 nanosheet during the cycling process between 14000 and 15000 s.

Table S1 Performance comparison of EC devices made up of WO_3 nanosheets

Samples	Transmittance change (%)	Switching response time		Long-term cycling stability (%)	Reference
		Coloration time (s)	Bleaching time (s)		
Small WO_3 nanosheets	41.78 (at 700 nm)	10.5	9.2	86 (1000 cycles)	This work
Thick WO_3 Flakes (20~50 nm)	46.19 (at 700 nm)	15.78	14.91	87 (1000 cycles)	Nano Lett. 2018, 18, 5646−5651
Thin WO_3 Flakes (<20 nm)	62.57 (at 700 nm)	10.74	6.97	94 (1000 cycles)	Nano Lett. 2018, 18, 5646−5651
$WO_3{\cdot}2H_2O$ ultrathin nanosheets	41.7 (at 700 nm)	9.7	5.1	-	Sci. Rep., 2013, 3, 1936
WO_3 nanosheets in bovine serum albumin	52.6 (at 1000 nm)	-		-	Adv.Mater.2017, 29, 1700326
$WO_3{\cdot}H_2O$ nanosheets without NH_4Cl	50.5 (at 633nm)	12.2	3.8	-	Sol. Energy Mater Sol. Cells 2018, 183, 59
$WO_3{\cdot}H_2O$ nanosheets with NH_4Cl	79.0 (at 633nm)	10.1	6.1	87.8 (2000 cycles)	Sol. Energy Mater Sol. Cells 2018, 183, 59
$WO_3{\cdot}0.33H_2O$ nanosheets with urea	67.1 (at 632.8nm)	21	8	-	RSC Adv. 2015, 5, 196
$WO_3{\cdot}0.33H_2O$ nanosheets without urea	55.8 (at 632.8nm)	36	8	-	RSC Adv. 2015, 5, 196
